# Microglial dynamics and emerging therapeutic strategies in CNS homeostasis and pathology

**DOI:** 10.3389/fphar.2025.1577809

**Published:** 2025-05-13

**Authors:** Jie Cao, Jianqing Yuan, Nanhai Liu, Kai Huang, Mingwei Guo

**Affiliations:** Department of Neurology, The First Affiliated Hospital of Gannan Medical University, Ganzhou, China

**Keywords:** microglial dynamics, therapeutic strategies, CNS homeostasis, pathology, microglia, CSF1, CSF1R, microglia depletion

## Abstract

Microglia, the resident immune cells of the central nervous system (CNS), are highly dynamic and play critical roles in maintaining CNS homeostasis. Under normal conditions, microglia continuously monitor their environment, clear cellular debris, and regulate homeostasis. In response to disease or injury, however, they undergo rapid morphological and functional changes, often adopting an amoeboid shape that facilitates phagocytosis of abnormal cells, pathogens, and external antigens. Microglia also proliferate in areas of injury or pathology, contributing to immune responses and tissue remodeling. Recently, pharmacological approaches targeting microglial depletion and repopulation have gained attention as a means to reset or modulate microglial function. Techniques such as CSF1R inhibition enable transient depletion of microglia, followed by rapid repopulation, potentially restoring homeostatic functions and mitigating chronic inflammation. This review explores the current understanding of microglial dynamics and highlights emerging therapeutic applications of microglial depletion and repopulation within the CNS.

## Introduction

Microglia are immune cells with a diverse array of essential functions in the central nervous system (CNS), including the retina (Huang et al., 2018a; Wang et al., 2023). Highly dynamic, microglia continuously monitor the CNS environment and play critical roles in synaptic pruning and phagocytosis, which are their primary functions (Wang et al., 2020; Parkhurst et al., 2013). During development, microglia refine neural circuits by pruning synapses (Zhao et al., 2015), while in pathological conditions, they phagocytose foreign antigens and dead cells (Alt et al., 2014). Phagocytosis is a hallmark of innate immune cell activation (Kalm et al., 2009).

Microglia possess an array of pattern recognition and immune receptors on their surface, enabling them to monitor neuronal activity, communicate with other cell types, and promote adaptive immune responses (Elmore et al., 2014). Furthermore, they are a major source of pro-inflammatory cytokines in the CNS. Unlike neurons, astrocytes, and oligodendrocytes, which have limited proliferative capacities, microglia can rapidly proliferate in response to CNS injury.

Microglia originate from the yolk sac in mice and exhibit dynamic changes in morphology and function throughout development and aging (Stanley and Chitu, 2014). In juvenile mice, microglia typically have a ramified, branched morphology and are primarily involved in synaptic pruning and immune surveillance (Chitu et al., 2016). In contrast, aged microglia exhibit a more amoeboid shape, shorter processes, and a pro-inflammatory phenotype, producing higher levels of inflammatory cytokines (Stanley et al., 1997; Elmore et al., 2014). They may also become less efficient at phagocytosis and debris clearance.

The microglial population adapts to physiological and pathological states in the CNS. During CNS injury, microglia rapidly increase in number (Stanley and Chitu, 2014). Colony-stimulating factor 1 receptor (CSF1R) is essential for their viability in the adult brain (Chitu et al., 2016). Knockout of the CSF1R gene significantly reduces microglial populations, while treatment with CSF1R inhibitors eliminates most resident microglia (Nakamichi et al., 2013). Upon cessation of CSF1R inhibitor treatment, microglia quickly repopulate and restore their presence in the brain. In this review, we will explore the mechanisms and implications of microglial repopulation in the CNS.

## Microglia depletion

Microglia are highly dynamic resident immune cells within the central nervous system (CNS), uniquely distinguished by their robust proliferative capacity compared to other CNS cell types (Huang et al., 2018a). As resident immune cells capable of rapid proliferation, microglia have emerged as a compelling target for therapeutic manipulation, sparking significant interest in their roles in CNS health and disease. Several strategies have been developed to deplete microglia in the CNS, including pharmacological approaches (Wang et al., 2023; Wang et al., 2020), genetic depletion (Parkhurst et al., 2013; Zhao et al., 2015), and irradiation (Alt et al., 2014; Kalm et al., 2009). Each method offers unique advantages and insights into microglial function and their potential for therapeutic interventions.

## Pharmacological depletion of microglia

The colony-stimulating factor 1 receptor (CSF1R) is a surface protein that serves as a critical regulator of myeloid lineage cells, including microglia and macrophages. Targeting CSF1R through pharmacological inhibitors has emerged as an effective strategy for depleting microglia in the CNS. By blocking CSF1R signaling, these inhibitors disrupt the survival and proliferation of microglia, leading to their rapid depletion. This approach has proven invaluable for studying microglial function and offers potential therapeutic avenues for resetting or modulating microglial activity in various CNS disorders (Elmore et al., 2014; Stanley and Chitu, 2014; Chitu et al., 2016). Both colony-stimulating factor 1 (CSF1) and interleukin-34 (IL34) serve as ligands for the colony-stimulating factor 1 receptor (CSF1R) CSF1R mediates critical signaling pathways that regulate the survival, proliferation, and differentiation of myeloid lineage cells, including microglia and macrophages (Stanley and Chitu, 2014; Stanley et al., 1997). These pathways are essential for maintaining microglial homeostasis and function, making CSF1R a pivotal target for therapeutic interventions aimed at modulating microglial activity in the CNS.

In the context of microglia, CSF1R plays a crucial role in maintaining their homeostasis in the CNS. Targeting CSF1R with specific inhibitors has proven to be an effective strategy for depleting microglia, enabling researchers to investigate their roles and contributions to various neurological conditions. Small-molecule CSF1R inhibitors, such as PLX5622 and PLX3397, have been widely used in animal models to achieve selective and reversible depletion of microglia. These inhibitors allow for precise temporal control, making them invaluable tools for studying microglial dynamics and their impact on CNS health and disease (Liddelow et al., 2017; Wang et al., 2019; De et al., 2014). By blocking CSF1R signaling, these inhibitors selectively induce microglial apoptosis without significantly affecting other CNS cells. This targeted approach has been instrumental in studies exploring microglial function and neuroinflammatory processes, providing valuable insights into the consequences of microglial depletion and subsequent repopulation on CNS health and disease.

Among the commonly used CSF1R inhibitors, PLX5622 and PLX3397 effectively deplete microglia, but they differ in specificity. PLX5622 exhibits higher specificity for CSF1R, minimizing off-target effects. In contrast, PLX3397 has broader activity, targeting other kinases such as KIT and FLT3, which may lead to more side effects.

**FIGURE 1:**
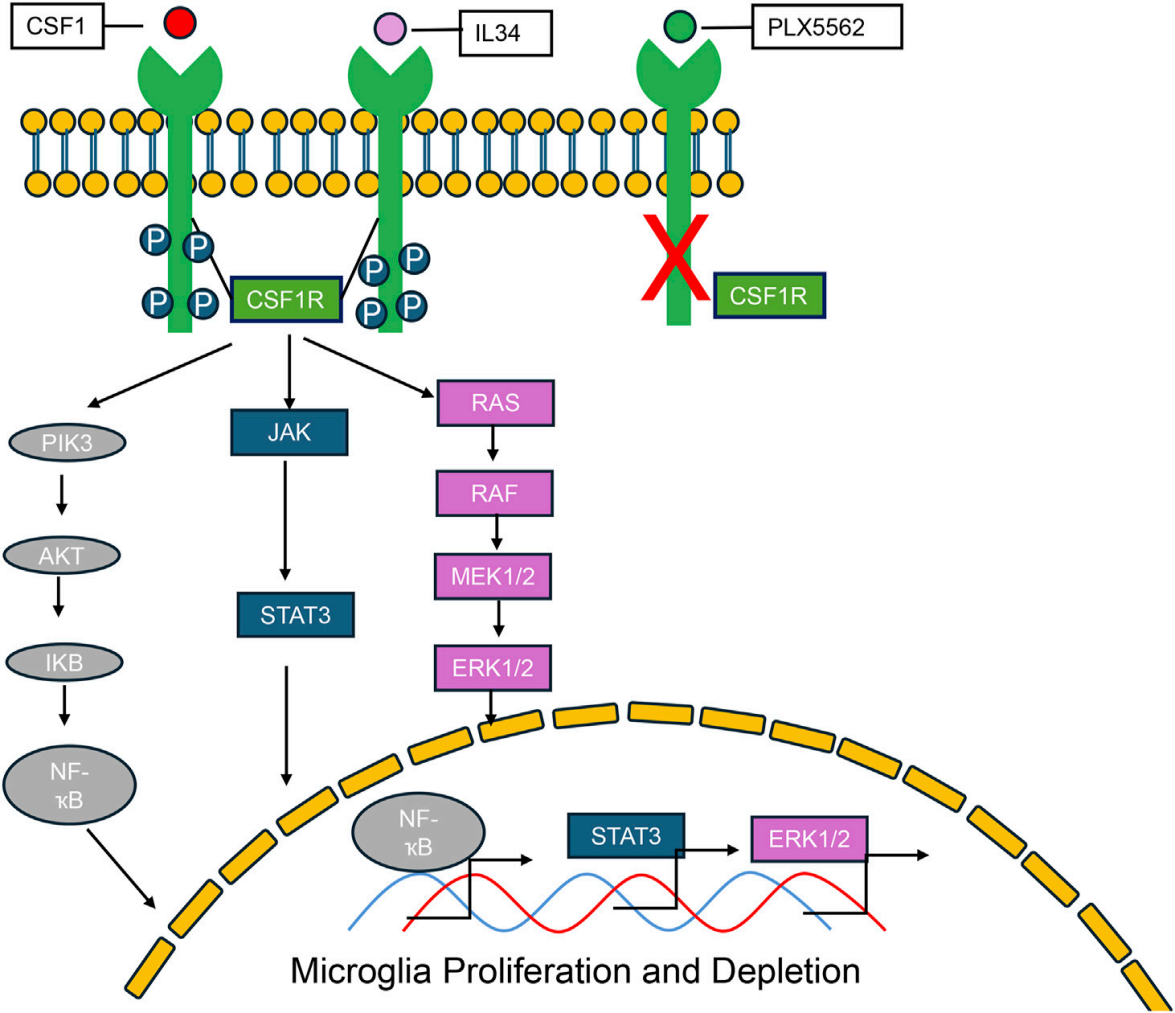
CSF-1R signaling pathways and the effects of CSF-1R inhibitors. Colony-stimulating factor 1 receptor (CSF-1R) is a shared receptor for both colony-stimulating factor 1 (CSF-1) and interleukin-34 (IL-34). Upon ligand binding, CSF-1R activates a cascade of downstream signaling pathways, including PI3K-AKT, ERK1/2, JAK/STAT, and NF-κB. These pathways regulate key cellular processes such as proliferation, survival, differentiation, and inflammatory responses, particularly within myeloid lineage cells. PLX5622, a selective small-molecule CSF-1R inhibitor, effectively blocks CSF-1R signaling, thereby suppressing the activation of these downstream pathways and reducing the survival and function of CSF-1R–dependent cells.

Upon ligand binding—either CSF1 or IL-34—the CSF1 receptor (CSF1R), a receptor tyrosine kinase, undergoes dimerization and autophosphorylation (Stanley and Chitu). This activation initiates a cascade of intracellular signaling events critical for myeloid cell survival, proliferation, and differentiation (Figure 1) (Stanley and Chitu, 2014; Nakamichi et al., 2013; Freuchet et al., 2021). The major downstream pathways include: PI3K–AKT signaling, which promotes cell survival and metabolic activity (Stanley and Chitu, 2014; Kelley et al., 1999; Murray et al., 2000; Chang et al., 2009); MAPK–ERK signaling, associated with cell proliferation and inflammatory responses (Lee, 2011; Richardson et al., 2015; Yao et al., 2005; Murga-Zamalloa et al., 2020; Chen et al., 2021); JAK–STAT signaling, mediating transcriptional activation of genes involved in immune function; Src-family kinase signaling, influencing cytoskeletal organization and cellular migration (Murga-Zamalloa et al., 2020; Truong et al., 2018); NF-κB activation, driving the expression of pro-inflammatory cytokines and chemokines (Lee, 2011; Caescu et al., 2015). Pharmacological inhibitors targeting CSF1R (PLX3397and BLZ945) effectively block these downstream pathways by preventing receptor activation (Horti et al., 2019; Chadarevian et al., 2023). In addition, pathway-specific inhibitors—such as LY294002 for PI3K, U0126 for MEK/ERK, and Ruxolitinib for JAK/STAT—have been widely used to dissect the individual contributions of these branches to CSF1R-driven cellular outcomes (Stanley and Chitu, 2014; Richardson et al., 2015; Sampaio et al., 2011; Karki et al., 2014; Sehgal et al., 2018).

Despite its specificity, CSF1R inhibition with PLX5622 extends beyond microglial depletion. It also significantly impacts circulating and tissue macrophages, with long-term effects persisting even after the cessation of treatment. These findings underscore the broader implications of CSF1R inhibition and highlight the need to carefully consider both short- and long-term effects when using these inhibitors in research and therapeutic contexts (Lei et al., 2020). CSF1R inhibition not only depletes microglia but also induces lasting changes in bone marrow-derived macrophages. Notably, it reduces interleukin-1β levels, CD68 expression, and phagocytic activity while sparing CD208 expression following endotoxin exposure (Elmore et al., 2014). These findings highlight the broader immunological impacts of CSF1R inhibitors, demonstrating their ability to modulate macrophage functionality and inflammatory responses beyond the CNS (Elmore et al., 2014).

## Genetic depletion microglia

A key limitation of small-molecule drugs for microglial depletion, such as CSF1R inhibitors, is their unintended impact on other myeloid cells beyond microglia (Bosch et al., 2023; Tan et al., 2021; Bohannon et al., 2024). These off-target effects can result in systemic immune modulation, including long-term changes in bone marrow-derived macrophages, such as altered cytokine expression and reduced phagocytic activity, which may persist even after treatment cessation (Lei et al., 2020).

## Irradiation

Cranial irradiation is a common and widely used treatment modality for managing cancers, particularly those involving the brain or with a high risk of metastasis to the central nervous system (CNS). This technique delivers targeted doses of radiation to eliminate cancerous cells, reduce tumor size, and prevent further disease progression. Despite its efficacy in controlling cancer, cranial irradiation often comes with significant side effects, including neurocognitive decline, inflammation, and damage to healthy brain tissue. Understanding the cellular and molecular impacts of cranial irradiation on the CNS is critical for improving therapeutic outcomes and minimizing adverse effects (Pazzaglia et al., 2020; Delaney et al., 2005; Moding et al., 2013). However, microglia become highly activated following cranial irradiation of the brain. This activation is characterized by morphological changes, increased expression of pro-inflammatory cytokines, and enhanced phagocytic activity. While microglial activation plays a role in the repair and clearance of damaged cells, chronic or excessive activation can contribute to neuroinflammation, exacerbating radiation-induced damage to healthy brain tissue. The persistent activation of microglia following cranial irradiation is a significant factor in the development of neurocognitive impairments and other long-term side effects, underscoring the need for strategies to modulate microglial responses in this context (Chiang et al., 1993; Kyrkanides et al., 1999). Cognitive dysfunction is a well-documented side effect of cranial irradiation of the brain. Microglial activation has been strongly associated with this cognitive decline, as activated microglia can exacerbate neuroinflammation and disrupt neural circuits critical for memory and learning. Studies have shown that depleting activated microglia can attenuate cognitive dysfunction following cranial irradiation, highlighting their role in mediating these adverse effects (Acharya et al., 2016). In addition to microglial activation, cranial irradiation also impairs the proliferative capacity of neural progenitor cells in the brain. This disruption affects neurogenesis, particularly in the hippocampus, a brain region essential for cognitive functions. The combined effects of microglial activation and reduced neurogenesis contribute to the long-term cognitive deficits observed after cranial irradiation. These findings emphasize the importance of targeting microglial activation and supporting neural regeneration to mitigate the cognitive side effects of this treatment.

The number of microglia decreases significantly after cranial irradiation (Kalm et al., 2009; Strohm et al., 2024; Chen et al., 2016; Eriksson and Stigbrand, 2010; Xue et al., 2014; Mizumatsu et al., 2003; Moravan et al., 2011). The primary cause of this microglial loss is DNA damage induced by irradiation (Menzel et al., 2018; Gutierrez-Quintana et al., 2022). DNA damage impairs the survival of microglia, leading to apoptosis and a reduction in their population.

Even in the young brain, which typically has robust regenerative and repair mechanisms, irradiation significantly reduces microglial numbers (Kalm et al., 2009; Han et al., 2016). Even in the young brain, which typically has robust regenerative and repair mechanisms, irradiation significantly reduces microglial numbers.

## Microglia repopulation

Both genetic and pharmacological methods for depleting microglia are effective approaches for studying their roles in the CNS (Wang and Cepko, 2022; Basilico et al., 2022). However, pharmacological depletion of microglia has broader applicability in research due to its ease of use and flexibility (Han et al., 2017; Barnett et al., 2021; Graykowski and Cudaback, 2021). Pharmacological methods, such as the use of CSF1R inhibitors, are particularly convenient for testing and therapeutic applications across various CNS disease models (Wang et al., 2023; Spangenberg et al., 2019; Sosna et al., 2018; Ortega-Martinez et al., 2019). These approaches allow for precise temporal control over microglial depletion and repopulation, making them a versatile tool for investigating microglial function and exploring potential treatments for neurodegenerative diseases, neuroinflammation, and other CNS disorders.

Microglia are highly self-regulating cells, constantly monitoring changes in the CNS under physiological conditions (Prinz et al., 2019). Studies have demonstrated that microglial populations recover rapidly after depletion (Huang et al., 2018a; Rice et al., 2017). In the brain, the source of repopulated microglia is derived from the surviving microglia that persist following depletion (Huang et al., 2018a). In contrast, the repopulation of microglia in the retina originates from two distinct sources (Huang et al., 2018b). This difference in the sources of repopulated microglia between the brain and retina highlights the unique dynamics of microglial recovery in these regions. Further research is needed to understand the underlying mechanisms driving these differences and their implications for CNS and retinal health and disease.

Combining cell lineage tracing with bone marrow cell transplantation offers a powerful approach to monitor the source of repopulated microglia (Xu et al., 2020; Cuadros et al., 2022). This method enables precise tracking of microglial origin and dynamics, providing critical insights into the mechanisms of microglial repopulation. Understanding the detailed process of microglial repopulation remains an area of great interest, as it could shed light on the pathways and signals driving microglial recovery.

Both microglia and macrophages belong to the myeloid cell lineage and share several marker genes, such as Cx3cr1, Cd68, and Trem2 (Hopperton et al., 2018; Wang et al., 2024; Greter et al., 2015). This overlap raises the intriguing possibility that macrophages, under certain conditions, could enter the CNS and potentially replace microglia. Such a replacement could open new avenues for treating CNS diseases by leveraging the plasticity and regenerative capabilities of macrophages. Exploring this possibility and the functional integration of macrophages into the CNS microenvironment warrants further investigation, as it could provide innovative strategies for modulating immune responses in the CNS.

**FIGURE 2:**
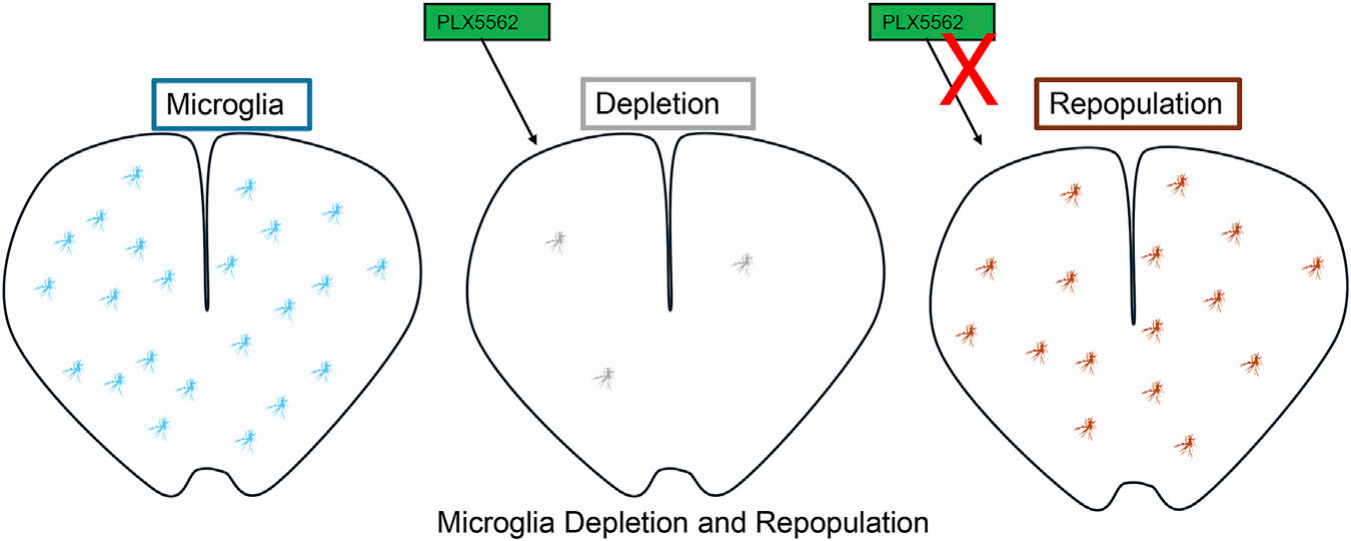
Microglial Depletion and Repopulation. Treatment with PLX5622, a CSF-1R inhibitor, leads to efficient depletion of microglia. Upon withdrawal of PLX5622, microglia undergo rapid repopulation, restoring their numbers and distribution within the central nervous system.

We hypothesize that microglial repopulation following depletion is regulated by CSF1–CSF1R signaling, which activates downstream pathways including PI3K–AKT, MAPK–ERK, and JAK–STAT to promote the survival, proliferation, and differentiation of repopulating microglial precursors. Furthermore, modulation of these pathways through pharmacological inhibitors or genetic manipulation may alter the dynamics, efficiency, and functional phenotypes of repopulating microglia (Figure 2).

## Applications and challenges

Microglia play a crucial role in maintaining CNS environmental stability and homeostasis (Gao et al., 2023; Colonna and Butovsky, 2017; Michell-Robinson et al., 2015). However, abnormally activated microglia can accelerate the progression of various neurological diseases (Bachiller et al., 2018). Therefore, distinguishing between “good” microglia, which support CNS health, and “bad” microglia, which contribute to disease development, is of great importance *in vivo*. Strategies aimed at repopulating “good” microglia while eliminating “bad” microglia may offer a novel therapeutic approach for treating CNS-related diseases such as Alzheimer’s disease (AD) and Parkinson’s disease (PD).

Given the vital role of microglia in CNS function and the potential risks associated with their manipulation, any therapeutic intervention involving microglia in humans requires careful consideration and thorough evaluation (Colonna and Butovsky, 2017; Cartier et al., 2014; Rock et al., 2004). Balancing the benefits of targeting microglia with the potential for unintended consequences is essential to ensure the safety and efficacy of such treatments.

## Summary

Microglia, often described as a double-edged sword, play a pivotal role in the progression and resolution of CNS diseases (Liu et al., 2021). Identifying “good microglia,” which promote CNS health, and “bad microglia,” which exacerbate disease, is essential for understanding their functions *in vivo*. Furthermore, specifically replacing “bad microglia” with “good microglia” in CNS diseases holds potential for mitigating disease progression.

As the immune cells of the CNS, microglia exhibit a remarkable capacity for repopulation following pharmacological depletion (Najafi et al., 2018; Han et al., 2019). Modulating the microglial population—either by selectively enhancing the presence of beneficial microglia or by depleting detrimental ones—offers a promising therapeutic strategy for treating brain diseases and retinopathies. This approach could pave the way for targeted interventions aimed at restoring CNS homeostasis and alleviating disease burden.

## References

[B1] Acharya M. M. Green K. N. Allen B. D. Najafi A. R. Syage A. Minasyan H. (2016). Elimination of microglia improves cognitive function following cranial irradiation. Sci. Rep. 6, 31545. 10.1038/srep31545 27516055 PMC4981848

[B2] Alt C. Runnels J. M. Mortensen L. J. Zaher W. Lin C. P. (2014). *In vivo* imaging of microglia turnover in the mouse retina after ionizing radiation and dexamethasone treatment. Invest Ophthalmol. Vis. Sci. 55, 5314–5319. 10.1167/iovs.14-14254 25082884

[B3] Bachiller S. Jimenez-Ferrer I. Paulus A. Yang Y. Swanberg M. Deierborg T. (2018). Microglia in neurological diseases: a road map to brain-disease dependent-inflammatory response. Front. Cell Neurosci. 12, 488. 10.3389/fncel.2018.00488 30618635 PMC6305407

[B4] Barnett A. M. Crews F. T. Coleman L. G. (2021). Microglial depletion and repopulation: a new era of regenerative medicine? Neural Regen. Res. 16, 1204–1205. 10.4103/1673-5374.300439 33269777 PMC8224128

[B5] Basilico B. Ferrucci L. Khan A. Di Angelantonio S. Ragozzino D. Reverte I. (2022). What microglia depletion approaches tell us about the role of microglia on synaptic function and behavior. Front. Cell Neurosci. 16, 1022431. 10.3389/fncel.2022.1022431 36406752 PMC9673171

[B6] Bohannon D. G. Zablocki-Thomas L. D. Leung E. S. Dupont J. K. Hattler J. B. Kowalewska J. (2024). CSF1R inhibition depletes brain macrophages and reduces brain virus burden in SIV-infected macaques. Brain 147, 3059–3069. 10.1093/brain/awae153 39049445 PMC11370798

[B7] Bosch A. J. T. Keller L. Steiger L. Rohm T. V. Wiedemann S. J. Low A. J. Y. (2023). CSF1R inhibition with PLX5622 affects multiple immune cell compartments and induces tissue-specific metabolic effects in lean mice. Diabetologia 66, 2292–2306. 10.1007/s00125-023-06007-1 37792013 PMC10627931

[B8] Caescu C. I. Guo X. Tesfa L. Bhagat T. D. Verma A. Zheng D. (2015). Colony stimulating factor-1 receptor signaling networks inhibit mouse macrophage inflammatory responses by induction of microRNA-21. Blood 125, e1–e13. 10.1182/blood-2014-10-608000 25573988 PMC4335087

[B9] Cartier N. Lewis C. A. Zhang R. Rossi F. M. (2014). The role of microglia in human disease: therapeutic tool or target? Acta Neuropathol. 128, 363–380. 10.1007/s00401-014-1330-y 25107477 PMC4131134

[B10] Chadarevian J. P. Lombroso S. I. Peet G. C. Hasselmann J. Tu C. Marzan D. E. (2023). Engineering an inhibitor-resistant human CSF1R variant for microglia replacement. J. Exp. Med. 220 (3), 2202023. 10.1084/jem.20220857 PMC9814156 36584406

[B11] Chang M. Hamilton J. A. Scholz G. M. Masendycz P. Macaulay S. L. Elsegood C. L. (2009). Phosphatidylinostitol-3 kinase and phospholipase C enhance CSF-1-dependent macrophage survival by controlling glucose uptake. Cell. Signal. 21, 1361–1369. 10.1016/j.cellsig.2009.04.003 19376223

[B12] Chen H. Chong Z. Z. De Toledo S. M. Azzam E. I. Elkabes S. Souayah N. (2016). Delayed activation of human microglial cells by high dose ionizing radiation. Brain Res. 1646, 193–198. 10.1016/j.brainres.2016.06.002 27265419

[B13] Chen M. J. Ramesha S. Weinstock L. D. Gao T. Ping L. Xiao H. (2021). Extracellular signal-regulated kinase regulates microglial immune responses in Alzheimer's disease. J. Neurosci. Res. 99, 1704–1721. 10.1002/jnr.24829 33729626 PMC8919593

[B14] Chiang C. S. McBride W. H. Withers H. R. (1993). Radiation-induced astrocytic and microglial responses in mouse brain. Radiother. Oncol. 29, 60–68. 10.1016/0167-8140(93)90174-7 8295989

[B15] Chitu V. Gokhan S. Nandi S. Mehler M. F. Stanley E. R. (2016). Emerging roles for CSF-1 receptor and its ligands in the nervous system. Trends Neurosci. 39, 378–393. 10.1016/j.tins.2016.03.005 27083478 PMC4884457

[B16] Colonna M. Butovsky O. (2017). Microglia function in the central nervous system during health and neurodegeneration. Annu. Rev. Immunol. 35, 441–468. 10.1146/annurev-immunol-051116-052358 28226226 PMC8167938

[B17] Cuadros M. A. Sepulveda M. R. Martin-Oliva D. Marin-Teva J. L. Neubrand V. E. (2022). Microglia and microglia-like cells: similar but different. Front. Cell Neurosci. 16, 816439. 10.3389/fncel.2022.816439 35197828 PMC8859783

[B18] De I. Nikodemova M. Steffen M. D. Sokn E. Maklakova V. I. Watters J. J. (2014). CSF1 overexpression has pleiotropic effects on microglia *in vivo* . Glia 62, 1955–1967. 10.1002/glia.22717 25042473 PMC4205273

[B19] Delaney G. Jacob S. Featherstone C. Barton M. (2005). The role of radiotherapy in cancer treatment: estimating optimal utilization from a review of evidence-based clinical guidelines. Cancer 104, 1129–1137. 10.1002/cncr.21324 16080176

[B20] Elmore M. R. Najafi A. R. Koike M. A. Dagher N. N. Spangenberg E. E. Rice R. A. (2014). Colony-stimulating factor 1 receptor signaling is necessary for microglia viability, unmasking a microglia progenitor cell in the adult brain. Neuron 82, 380–397. 10.1016/j.neuron.2014.02.040 24742461 PMC4161285

[B21] Eriksson D. Stigbrand T. (2010). Radiation-induced cell death mechanisms. Tumour Biol. 31, 363–372. 10.1007/s13277-010-0042-8 20490962

[B22] Freuchet A. Salama A. Remy S. Guillonneau C. Anegon I. (2021). IL-34 and CSF-1, deciphering similarities and differences at steady state and in diseases. J. Leukoc. Biol. 110, 771–796. 10.1002/JLB.3RU1120-773R 33600012

[B23] Gao C. Jiang J. Tan Y. Chen S. (2023). Microglia in neurodegenerative diseases: mechanism and potential therapeutic targets. Signal Transduct. Target Ther. 8, 359. 10.1038/s41392-023-01588-0 37735487 PMC10514343

[B24] Graykowski D. Cudaback E. (2021). Don't know what you got till it's gone: microglial depletion and neurodegeneration. Neural Regen. Res. 16, 1921–1927. 10.4103/1673-5374.308078 33642360 PMC8343303

[B25] Greter M. Lelios I. Croxford A. L. (2015). Microglia versus myeloid cell nomenclature during brain inflammation. Front. Immunol. 6, 249. 10.3389/fimmu.2015.00249 26074918 PMC4443742

[B26] Gutierrez-Quintana R. Walker D. J. Williams K. J. Forster D. M. Chalmers A. J. (2022). Radiation-induced neuroinflammation: a potential protective role for poly(ADP-ribose) polymerase inhibitors? Neurooncol Adv. 4, vdab190. 10.1093/noajnl/vdab190 35118383 PMC8807076

[B27] Han J. Harris R. A. Zhang X. M. (2017). An updated assessment of microglia depletion: current concepts and future directions. Mol. Brain 10 (25), 25. 10.1186/s13041-017-0307-x 28629387 PMC5477141

[B28] Han J. Zhu K. Zhang X. M. Harris R. A. (2019). Enforced microglial depletion and repopulation as a promising strategy for the treatment of neurological disorders. Glia 67, 217–231. 10.1002/glia.23529 30378163 PMC6635749

[B29] Han W. Umekawa T. Zhou K. Zhang X. M. Ohshima M. Dominguez C. A. (2016). Cranial irradiation induces transient microglia accumulation, followed by long-lasting inflammation and loss of microglia. Oncotarget 7, 82305–82323. 10.18632/oncotarget.12929 27793054 PMC5347693

[B30] Hopperton K. E. Mohammad D. Trepanier M. O. Giuliano V. Bazinet R. P. (2018). Markers of microglia in post-mortem brain samples from patients with Alzheimer's disease: a systematic review. Mol. Psychiatry 23, 177–198. 10.1038/mp.2017.246 29230021 PMC5794890

[B31] Horti A. G. Naik R. Foss C. A. Minn I. Misheneva V. Du Y. (2019). PET imaging of microglia by targeting macrophage colony-stimulating factor 1 receptor (CSF1R). Proc. Natl. Acad. Sci. U. S. A. 116, 1686–1691. 10.1073/pnas.1812155116 30635412 PMC6358677

[B32] Huang Y. Xu Z. Xiong S. Qin G. Sun F. Yang J. (2018). Dual extra-retinal origins of microglia in the model of retinal microglia repopulation. Cell Discov. 4 (9), 9. 10.1038/s41421-018-0011-8 29507754 PMC5827656

[B33] Huang Y. Xu Z. Xiong S. Sun F. Qin G. Hu G. (2018). Repopulated microglia are solely derived from the proliferation of residual microglia after acute depletion. Nat. Neurosci. 21, 530–540. 10.1038/s41593-018-0090-8 29472620

[B34] Kalm M. Lannering B. Bjork-Eriksson T. Blomgren K. (2009). Irradiation-induced loss of microglia in the young brain. J. Neuroimmunol. 206, 70–75. 10.1016/j.jneuroim.2008.11.002 19070908

[B35] Karki P. Webb A. Zerguine A. Choi J. Son D. S. Lee E. (2014). Mechanism of raloxifene-induced upregulation of glutamate transporters in rat primary astrocytes. Glia 62, 1270–1283. 10.1002/glia.22679 24782323 PMC4061260

[B36] Kelley T. W. Graham M. M. Doseff A. I. Pomerantz R. W. Lau S. M. Ostrowski M. C. (1999). Macrophage colony-stimulating factor promotes cell survival through Akt/protein kinase B. J. Biol. Chem. 274, 26393–26398. 10.1074/jbc.274.37.26393 10473597

[B37] Kyrkanides S. Olschowka J. A. Williams J. P. Hansen J. T. O'Banion M. K. (1999). TNF alpha and IL-1beta mediate intercellular adhesion molecule-1 induction via microglia-astrocyte interaction in CNS radiation injury. J. Neuroimmunol. 95, 95–106. 10.1016/s0165-5728(98)00270-7 10229119

[B38] Lee A. W. (2011). The role of atypical protein kinase C in CSF-1-dependent Erk activation and proliferation in myeloid progenitors and macrophages. PloS One 6, e25580. 10.1371/journal.pone.0025580 22028782 PMC3196503

[B39] Lei F. Cui N. Zhou C. Chodosh J. Vavvas D. G. Paschalis E. I. (2020). CSF1R inhibition by a small-molecule inhibitor is not microglia specific; affecting hematopoiesis and the function of macrophages. Proc. Natl. Acad. Sci. U. S. A. 117, 23336–23338. 10.1073/pnas.1922788117 32900927 PMC7519218

[B40] Liddelow S. A. Guttenplan K. A. Clarke L. E. Bennett F. C. Bohlen C. J. Schirmer L. (2017). Neurotoxic reactive astrocytes are induced by activated microglia. Nature 541, 481–487. 10.1038/nature21029 28099414 PMC5404890

[B41] Liu J. Liu L. Wang X. Jiang R. Bai Q. Wang G. (2021). Microglia: a double-edged sword in intracerebral hemorrhage from basic mechanisms to clinical research. Front. Immunol. 12, 675660. 10.3389/fimmu.2021.675660 34025674 PMC8135095

[B42] Menzel F. Kaiser N. Haehnel S. Rapp F. Patties I. Schöneberg N. (2018). Impact of X-irradiation on microglia. Glia 66, 15–33. 10.1002/glia.23239 29024033

[B43] Michell-Robinson M. A. Touil H. Healy L. M. Owen D. R. Durafourt B. A. Bar-Or A. (2015). Roles of microglia in brain development, tissue maintenance and repair. Brain 138, 1138–1159. 10.1093/brain/awv066 25823474 PMC5963417

[B44] Mizumatsu S. Monje M. L. Morhardt D. R. Rola R. Palmer T. D. Fike J. R. (2003). Extreme sensitivity of adult neurogenesis to low doses of X-irradiation. Cancer Res. 63, 4021–4027. 12874001

[B45] Moding E. J. Kastan M. B. Kirsch D. G. (2013). Strategies for optimizing the response of cancer and normal tissues to radiation. Nat. Rev. Drug Discov. 12, 526–542. 10.1038/nrd4003 23812271 PMC3906736

[B46] Moravan M. J. Olschowka J. A. Williams J. P. O'Banion M. K. (2011). Cranial irradiation leads to acute and persistent neuroinflammation with delayed increases in T-cell infiltration and CD11c expression in C57BL/6 mouse brain. Radiat. Res. 176, 459–473. 10.1667/rr2587.1 21787181 PMC3191189

[B47] Murga-Zamalloa C. Rolland D. C. M. Polk A. Wolfe A. Dewar H. Chowdhury P. (2020). Colony-stimulating factor 1 receptor (CSF1R) activates AKT/mTOR signaling and promotes T-cell lymphoma viability. Clin. cancer Res. official J. Am. Assoc. Cancer Res. 26, 690–703. 10.1158/1078-0432.CCR-19-1486 PMC7002219 31636099

[B48] Murray J. T. Craggs G. Wilson L. Kellie S. (2000). Mechanism of phosphatidylinositol 3-kinase-dependent increases in BAC1.2F5 macrophage-like cell density in response to M-CSF: phosphatidylinositol 3-kinase inhibitors increase the rate of apoptosis rather than inhibit DNA synthesis. Inflamm. Res. 49, 610–618. 10.1007/s000110050638 11131301

[B49] Najafi A. R. Crapser J. Jiang S. Ng W. Mortazavi A. West B. L. (2018). A limited capacity for microglial repopulation in the adult brain. Glia 66, 2385–2396. 10.1002/glia.23477 30370589 PMC6269202

[B50] Nakamichi Y. Udagawa N. Takahashi N. (2013). IL-34 and CSF-1: similarities and differences. J. bone mineral metabolism 31, 486–495. 10.1007/s00774-013-0476-3 23740288

[B51] Ortega-Martinez S. Palla N. Zhang X. Lipman E. Sisodia S. S. (2019). Deficits in enrichment-dependent neurogenesis and enhanced anxiety behaviors mediated by expression of Alzheimer's disease-linked Ps1 variants are rescued by microglial depletion. J. Neurosci. 39, 6766–6780. 10.1523/JNEUROSCI.0884-19.2019 31217332 PMC6703877

[B52] Parkhurst C. N. Yang G. Ninan I. Savas J. N. Yates J. R. 3rd Lafaille J. J. (2013). Microglia promote learning-dependent synapse formation through brain-derived neurotrophic factor. Cell 155, 1596–1609. 10.1016/j.cell.2013.11.030 24360280 PMC4033691

[B53] Pazzaglia S. Briganti G. Mancuso M. Saran A. (2020). Neurocognitive decline following radiotherapy: mechanisms and therapeutic implications. Cancers (Basel) 12. 10.3390/cancers12010146 PMC7017115 31936195

[B54] Prinz M. Jung S. Priller J. (2019). Microglia biology: one century of evolving concepts. Cell 179, 292–311. 10.1016/j.cell.2019.08.053 31585077

[B55] Rice R. A. Pham J. Lee R. J. Najafi A. R. West B. L. Green K. N. (2017). Microglial repopulation resolves inflammation and promotes brain recovery after injury. Glia 65, 931–944. 10.1002/glia.23135 28251674 PMC5395311

[B56] Richardson E. T. Shukla S. Nagy N. Boom W. H. Beck R. C. Zhou L. (2015). ERK signaling is essential for macrophage development. PloS One 10, e0140064. 10.1371/journal.pone.0140064 26445168 PMC4596867

[B57] Rock R. B. Gekker G. Hu S. Sheng W. S. Cheeran M. Lokensgard J. R. (2004). Role of microglia in central nervous system infections. Clin. Microbiol. Rev. 17, 942–964. 10.1128/CMR.17.4.942-964.2004 15489356 PMC523558

[B58] Sampaio N. G. Yu W. Cox D. Wyckoff J. Condeelis J. Stanley E. R. (2011). Phosphorylation of CSF-1R Y721 mediates its association with PI3K to regulate macrophage motility and enhancement of tumor cell invasion. J. cell Sci. 124, 2021–2031. 10.1242/jcs.075309 21610095 PMC3104034

[B59] Sehgal A. Donaldson D. S. Pridans C. Sauter K. A. Hume D. A. Mabbott N. A. (2018). The role of CSF1R-dependent macrophages in control of the intestinal stem-cell niche. Nat. Commun. 9, 1272. 10.1038/s41467-018-03638-6 29593242 PMC5871851

[B60] Sosna J. Philipp S. Albay R. 3rd Reyes-Ruiz J. M. Baglietto-Vargas D. LaFerla F. M. (2018). Early long-term administration of the CSF1R inhibitor PLX3397 ablates microglia and reduces accumulation of intraneuronal amyloid, neuritic plaque deposition and pre-fibrillar oligomers in 5XFAD mouse model of Alzheimer's disease. Mol. Neurodegener. 13 (11), 11. 10.1186/s13024-018-0244-x 29490706 PMC5831225

[B61] Spangenberg E. Severson P. L. Hohsfield L. A. Crapser J. Zhang J. Burton E. A. (2019). Sustained microglial depletion with CSF1R inhibitor impairs parenchymal plaque development in an Alzheimer's disease model. Nat. Commun. 10, 3758. 10.1038/s41467-019-11674-z 31434879 PMC6704256

[B62] Stanley E. R. Berg K. L. Einstein D. B. Lee P. S. Pixley F. J. Wang Y. (1997). Biology and action of colony--stimulating factor-1. Mol. Reprod. Dev. 46, 4–10. 10.1002/(SICI)1098-2795(199701)46:1<4::AID-MRD2>3.0.CO;2-V 8981357

[B63] Stanley E. R. Chitu V. (2014). CSF-1 receptor signaling in myeloid cells. Cold Spring Harb. Perspect. Biol. 6. 10.1101/cshperspect.a021857 PMC4031967 24890514

[B64] Strohm A. O. Johnston C. Hernady E. Marples B. O'Banion M. K. Majewska A. K. (2024). Cranial irradiation disrupts homeostatic microglial dynamic behavior. J. Neuroinflammation 21 (82), 82. 10.1186/s12974-024-03073-z 38570852 PMC10993621

[B65] Tan I. L. Arifa R. D. N. Rallapalli H. Kana V. Lao Z. Sanghrajka R. M. (2021). CSF1R inhibition depletes tumor-associated macrophages and attenuates tumor progression in a mouse sonic Hedgehog-Medulloblastoma model. Oncogene 40, 396–407. 10.1038/s41388-020-01536-0 33159168 PMC7855734

[B66] Truong A. D. Hong Y. Lee J. Lee K. Kil D. K. Lillehoj H. S. (2018). Interleukin-34 regulates Th1 and Th17 cytokine production by activating multiple signaling pathways through CSF-1R in chicken cell lines. Int. J. Mol. Sci. 19 (6), 192018. 10.3390/ijms19061665 PMC6032434 29874806

[B67] Wang S. K. Cepko C. L. (2022). Targeting microglia to treat degenerative eye diseases. Front. Immunol. 13, 843558. 10.3389/fimmu.2022.843558 35251042 PMC8891158

[B68] Wang S. K. Xue Y. Cepko C. L. (2020). Microglia modulation by TGF-β1 protects cones in mouse models of retinal degeneration. J. Clin. Invest 130, 4360–4369. 10.1172/JCI136160 32352930 PMC7410072

[B69] Wang S. K. Xue Y. Rana P. Hong C. M. Cepko C. L. (2019). Soluble CX3CL1 gene therapy improves cone survival and function in mouse models of retinitis pigmentosa. Proc. Natl. Acad. Sci. U. S. A. 116, 10140–10149. 10.1073/pnas.1901787116 31036641 PMC6525490

[B70] Wang T. Kaneko S. Kriukov E. Alvarez D. Lam E. Wang Y. (2024). SOCS3 regulates pathological retinal angiogenesis through modulating SPP1 expression in microglia and macrophages. Mol. Ther. 32, 1425–1444. 10.1016/j.ymthe.2024.03.025 38504518 PMC11081920

[B71] Wang W. Li Y. Ma F. Sheng X. Chen K. Zhuo R. (2023). Microglial repopulation reverses cognitive and synaptic deficits in an Alzheimer's disease model by restoring BDNF signaling. Brain Behav. Immun. 113, 275–288. 10.1016/j.bbi.2023.07.011 37482204

[B72] Xu Z. Rao Y. Huang Y. Zhou T. Feng R. Xiong S. (2020). Efficient strategies for microglia replacement in the central nervous system. Cell Rep. 33, 108443. 10.1016/j.celrep.2020.108443 33238120

[B73] Xue J. Dong J. H. Huang G. D. Qu X. F. Wu G. Dong X. R. (2014). NF-κB signaling modulates radiation‑induced microglial activation. Oncol. Rep. 31, 2555–2560. 10.3892/or.2014.3144 24756575

[B74] Yao G. Q. Itokawa T. Paliwal I. Insogna K. (2005). CSF-1 induces fos gene transcription and activates the transcription factor Elk-1 in mature osteoclasts. Calcif. tissue Int. 76, 371–378. 10.1007/s00223-004-0099-8 15812575

[B75] Zhao L. Zabel M. K. Wang X. Ma W. Shah P. Fariss R. N. (2015). Microglial phagocytosis of living photoreceptors contributes to inherited retinal degeneration. EMBO Mol. Med. 7, 1179–1197. 10.15252/emmm.201505298 26139610 PMC4568951

